# Inter-Rater Reliability, Concurrent Validity and Sensitivity of Current Methods to Assess Trunk Function in Boccia Players with Cerebral Palsy

**DOI:** 10.3390/brainsci10030130

**Published:** 2020-02-26

**Authors:** Alba Roldan, David Barbado, Francisco J. Vera-Garcia, José M. Sarabia, Raul Reina

**Affiliations:** Sport Research Centre, Department of Sport Sciences, Miguel Hernández University, 03202 Elche, Spain; aroldan@umh.es (A.R.); dbarbado@umh.es (D.B.); fvera@umh.es (F.J.V.-G.); jsarabia@umh.es (J.M.S.)

**Keywords:** Paralympics, para-sport, classification, brain impairment, sitting balance, trunk control

## Abstract

Trunk function is a core factor to allocate Boccia players with cerebral palsy in BC1 and BC2 sport classes, according to the Boccia International Sports Federation (BISFed). However, the appropriateness of the current test to assess trunk function has never been studied to determine its reliability, validity and sensitivity to discriminate between different levels of impairment. Thirty-six players (BC1 = 13 and BC2 = 23) took part in this study. Trunk control was assessed through the BISFed trunk function scale (TFS) and a posturographic test battery consisting of two static and three dynamic tasks. The inter-rater reliability for the BISFed TFS was set at 94.44% of agreement. Moderate-to-high correlations were obtained between posturographic tasks (0.39 < r < 0.96; *p* < 0.05–0.01), while the BISFed TFS only correlated with two of the dynamic tasks and the overall dynamic score (−0.38 < r < −0.51; *p* < 0.05). The BISFed TFS was not able to discriminate between sport classes, whereas the static posturographic task did so (*p* = 0.004). Even though the current BISFed TFS presented good inter-rater reliability, it does not seem to have enough sensitivity to discriminate between BC1 and BC2. Although the static posturographic tasks were able to discriminate between sports classes, it seems necessary to develop new field tests assessing participants’ trunk stabilization abilities.

## 1. Introduction

Boccia is a Paralympic target sport played with soft leather balls, which requires precision and strong tactical skills. It offers sporting opportunities to individuals with severe physical impairments, including hypertonia, athetosis, ataxia, impaired muscle strength, impaired range of movement or limb deficiency [1]. Boccia players are classified into five sports classes, from BC1 to BC5 [1], according to how much impact a player’s impairment has on the performance of their throwing skills [2]. Specifically, BC1 and BC2 sport classes are for para-athletes who are “diagnosed with a neurological impairment affecting the central nervous system; spastic hypertonic quadriplegia or dyskinesia (athetosis/dystonia) or who may have a mixed picture including those with severe ataxia” [1]. This entails in significant coordination and trunk control limitations [3]. However, the descriptors provided by the classification rulebook [1] to discriminate between BC1 and BC2 sport classes according to their trunk control are qualitative and occasionally ambiguous. Therefore, from the author’s point of view, classifying players belonging to these two sport classes sometimes becomes a challenge. Consequently, it is essential to develop evidence-based classification to assess the real impact of players’ impairment on sports performance, allowing fair and equitable competition and avoiding unfair advantage caused by their degree of impairment.

In order to facilitate the classification process, the Boccia International Sports Federation (BISFed) classification rulebook [1] provides detailed guidelines on how the severity of the impairment limits players’ motor skills required to compete. Within these capabilities, trunk control is considered an essential aspect in this para-sport [1], as its deficit may hinder upper-extremities function related to the throwing success as grasping or carrying balls [4,5]. Current trunk control assessment relies on a field protocol (hereinafter “the BISFed trunk functional scale” (BISFed TFS)), due to its low cost, ease of performance and rapid implementation. This test evaluates, in a qualitative manner, players’ abilities to keep a stable sitting position performing the movement in sagittal, coronal and transverse planes, reaching as far as possible [1]. Although an objective classification is a basis for achieving a fair competition in Paralympic sport [2], the use of the BISFed TFS still raises doubts when used with classification purposes. First, there are no studies supporting the test validity (e.g., comparing it with a laboratory test), nor its ability to discriminate between sport classes (i.e., sensitivity) or the agreement among classifiers when using the test to evaluate the trunk function (i.e., inter-rater reliability). Second, the way that trunk control is assessed does not seem appropriate for the final purpose of the classification process, as the test intends to assess limit of stability rather than the ability of just maintaining sitting balance when moving in different planes or rotating the trunk, which differentiates between sport classes, especially between BC1 and BC2 players according to the current BISFed classification rulebook [1]. 

Given the above, the aims of this study are: (i) to study the inter-rater reliability among classifiers when using the current test that assesses trunk function in Boccia players, (ii) to study the concurrent validity of the BISFed TFS using a lab posturographic test battery as a gold standard and (iii) to explore the sensitivity of the Boccia TFS and the lab-based posturography to discriminate between players allocated in BC1 and BC2 sports classes (i.e., different levels of impairment or overall motor function).

## 2. Materials and Methods

### 2.1. Participants

A cross-sectional study was carried out with a group of 36 Boccia players with severe-to-moderate cerebral palsy (CP), from national and regional levels. Demographic information of the participants is provided in Table 1.

All participants met the following inclusion criteria: (i) have a diagnosis of CP; (ii) age 20–50 years; (iii) classified as BC1 (severe spastic, athetoid or mixed quadriplegia) or BC2 (moderate-to-severe spastic, athetoid or mixed quadriplegia) [1]; (iv) having a sports license to play Boccia; (v) no surgeries or Botulinum toxin-A injections in the six months prior to testing and (vi) able to follow the pertinent test instructions given by the researchers. The exclusion criteria were: (i) athletes classified as BC3, BC4 or BC5 or (ii) accompanied by comorbidity of intellectual impairment.

Participants were recruited from seven centers for people with cerebral palsy or related neurological conditions with sports programs, including the only Boccia club in the region. The chief researcher contacted all the centers to explain the aims of the study, the participants’ inclusion/exclusion criteria, the testing procedures and the time commitment. After receiving the willingness to participate in the study by the centers/club, the assessment dates were agreed individually. Additionally, a member of the research staff traveled in advance to check the inclusion/exclusion criteria of the potential participants and check players’ sport licenses to record their sport classes. The ethics approval was obtained through the local University Ethics Committee (Reference DPS-RVV-001-10). All participants gave written consent in accordance with the Declaration of Helsinki.

### 2.2. Procedure

Participants completed all the assessments in one single session (i.e., all participants performed the BISFed TFS and the posturographic test battery on the same day). To offer a familiar environment for participants, all the testing equipment was taken to each center/club. 

During the testing session, participants were classified according to the gross motor functional classification system (GMFCS) [6]. Subsequently, the BISFed TFS was carried out to assess players’ trunk function. Once both assessments were finished, participants were taken to another room to perform the posturographic protocol to evaluate their postural control in static and dynamic conditions. 

### 2.3. BISFed TFS 

**Figure 1:**
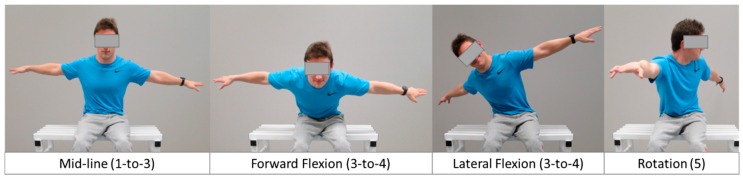
Movements required for scoring trunk function according to the Boccia International Sports Federation (BISFed) trunk function scale (TFS).

The BISFed TFS is a trunk test based on clinical expertise. This test was carried out following the current classification protocols [1]. Participants were sat upright on a bench and asked to lean away from the midline vertical position to the greatest distance in the sagittal (anterior-posterior displacement) and coronal (medial-lateral displacements) planes, without falling or reaching for support. This assessment also included trunk twist to evaluate trunk rotation capacity (see Figure 1). As in real classification, due to the severity of players’ impairment, some participants were assessed in their own wheelchairs because of their inability to keep their trunk upright without external aid (e.g., strapping or backrest support). The Boccia classification rulebook considers trunk function according to five qualitative items, following an ordinal basis from less (1) to more (5) trunk function: 1 = the athlete requires restraint to prevent falling out of the chair, 2 = the athlete uses head to center after throwing or a disturbance, 3 = the athlete uses arms/hands to center after throwing or a disturbance, 4 = the athlete can return to the upright position without the head/hands after throwing or a disturbance and 5 = the athlete presents good/fair trunk rotation. This ordinal checklist has been used as a 5-point ratio scale for this study (1 point = lowest trunk function to 5 = highest trunk function). Trunk function was assessed by two international classifiers, members of the BISFed Classification Committee (see Table 2), with the highest qualification level (i.e., Level 3 international Boccia classifier). They independently scored the participants´ trunk function according to the abovementioned 5-point ratio scale.

### 2.4. Posturographic Test Battery

**Figure 2:**
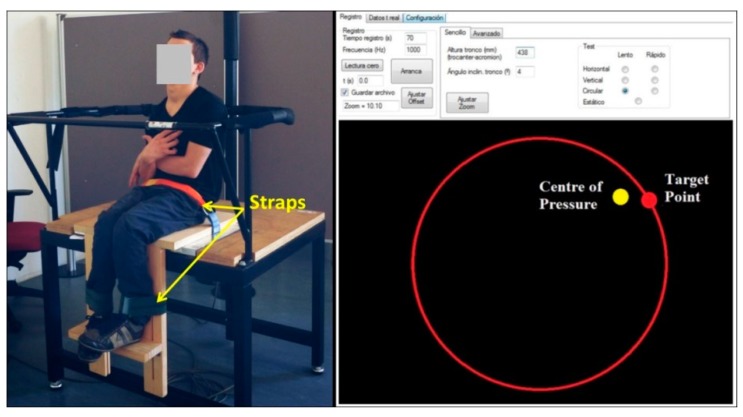
Participants set up on the stable surface (**left**). Screen feedback with the red dot representing the target and its trajectory and the yellow dot representing participants’ center of pressure (**right**).

Following the protocol by Barbado et al. [7,8], participants were seated in a wooden chair placed on a force platform (Kistler, Switzerland, Model 9286AA at 1000 sample/s), crossing their arms firmly in front of their chest or as close as possible and the hip and the legs strapped to the seat (90° knee flexion) (Figure 1). All tasks, except one, required that participants adjust his/her center of pressure (CoP) position according to a target point, which was represented by a red dot (radius = 60 mm), shown on a 106 × 138-cm screen and placed at 3.5 m in front of the participant. Real-time visual feedback of participant’s center of pressure (CoP) displacement was represented by a yellow dot (radius = 60 mm), also shown on the screen (see Figure 2). 

The posturographic test battery included five sitting conditions: two static tasks, one without (SNVF) and one with visual feedback (SVF) and three dynamic tasks with the visual feedback requiring medial-lateral (DML), anterior-posterior (DAP) and circular (DC) trunk displacements. In the static conditions without feedback, the CoP and the target point were not presented on the screen. In this task, participants were asked to maintain their upright sitting posture as still as possible. In the dynamic tasks, participants were instructed to follow the target point that moved at the rate of 0.05 Hz in medial-lateral, anterior-posterior and circular motions [7,8]. The amplitude of the target point displacement corresponded to a 4° inclination angle of the participants’ center of mass of the upper body [7].

Each task was performed twice, with a 70-s duration and a 60-s rest interval between trials. It was considered a successful trial when the participant completed the task with two or fewer instances of external assistance or ≤15 s of the 70-s total testing time. If successful, then the participant advanced to the next task. However, if the athlete was unable to complete the second trial of the task, his/her evaluation stopped at that stage, and it did not progress onto the following task. 

#### Posturography Data Reduction 

The CoP time series obtained from the force platform was filtered using a low-pass filter (4th-order, zero-phase-lag, Butterworth, 5 Hz cut-off frequency) [9]. There is little physiological significance to the CoP signal frequencies above 10 Hz [10], so the CoP time series were subsampled at 20 Hz. In addition, the first 10 s of each trial were discarded to avoid nonstationary behavior related to the beginning of the trial [11]. The mean radial error (MRE) was used as a global measure to quantify trunk performance during the trials. The MRE was calculated as the average of the vector distance magnitude (mm) of the CoP from the target point or the participant’s mean CoP position [12] for trials with and without visual feedback, respectively. The best trial performed for each condition (i.e., lowest values of MRE) was used for data analysis. A higher score for MRE indicates more trunk sway, showing a worse performance during testing.

Summing up, the static (SNVF + SVF) and the dynamic (DML + DAP + DC) composite scores were also reported as mean ± standard deviation. 

### 2.5. Statistical Analysis

#### 2.5.1. Inter-Rater Reliability

The degree of agreement among testers assessing with the BISFed TFS was used as a numerical estimation. The percentage of agreement was calculated by the fraction of the number of ratings in agreement with the total number of ratings (i.e., agreements + disagreements). A percentage of agreement above 90% is required to consider as a “high agreement” [13].

#### 2.5.2. Validity

A Wilcoxon-Mann Whitney test was applied for the GMFCS variable due to the nominal nature of this variable. The Hedges’ g index (dg) was used to calculate the effect sizes of between-group differences [14]. This index is based on Cohen’s d index [15] but provides an effect size estimation when reducing the bias caused by small samples (*n* < 20), such as the BC1 group in our study. Hedge’s g was interpreted as: large (dg > 0.8), moderate (0.5 < dg ≤ 0.8), small (0.2 < dg ≤ 0.5) and trivial (dg < 0.2). A Pearson test was used to analyze the correlations between the BISFed TFS and the posturographic test battery (r). The threshold values for the Pearson product-moment were used to construe the results: negligible (r ≤ 0.30), low (0.30 < r ≤ 0.50), moderate (0.50 < r ≤ 0.70), high (0.70 < r ≤ 0.90) and very high (0.90 < r ≤ 1.00) [16]. Finally, one-way ANOVA was conducted for all dependent variables to examine the differences between groups (BC1 vs. BC2 sport classes). 

#### 2.5.3. Sensitivity

Receiver operating characteristic (ROC) curve analysis was also used to evaluate the sensitivity of the BISFed TFS and the posturography to classify participants into BC1 or BC2 sport classes based on their trunk function impairment severity. The area under the ROC curve (AUC) was interpreting as excellent (0.9 < AUC < 1.0), good (0.8 < AUC < 0.9), worthless (0.7 < AUC < 0.8) or not good (0.6 < AUC < 0.7) [17]. Regarding posturography, the ROC analysis was calculated only for the static composite score (i.e., SNVF + SVF), since this variable grouped the two posturographic conditions that showed significant differences between sport classes. Afterwards, the MRE values obtained by the participants were linked to the sport classes. The optimal threshold point was determined by the Youden index to maximize both sensitivity and specificity indexes. ROC analyses were only applied in those parameters that showed significant differences between groups.

Data analyses were performed using the Statistical Package for Social Sciences (version 24 for Windows, SPSS Inc., Chicago, IL, USA). Statistical significance was set at an alpha level of *p* < 0.05.

## 3. Results

### 3.1. Inter-Rater Reliability 

Analyzing the inter-rater reliability of the BISFed TFS, an agreement of 94.44% between testers was reached. Only two classifications obtained discrepant results. Thus, it can be construed as a high percentage of agreement.

### 3.2. Concurrent Validity between BISFed TFS and Posturography

A Wilcoxon-Mann Whitney test was conducted for the GMFCS scores to highlight the different levels of impairment for the players belonging the BC1 and the BC2 classes, confirming that BC2 players had a better overall function (Mann-Whitney U = 78.00, W = 354.00, *z* = −2.90; *p* < 0.01, two-tailed; dg = 1.05, large). 

The Pearson product moment analysis indicated small-to-moderate negative correlations between the BISFed TFS and two of the dynamic posturographic tasks (DML: r = −0.38; *p* = 0.03 and DC: r = −0.46; *p* = 0.02), as well as the composite score of the dynamic conditions (r = −0.51; *p* < 0.01) (Table 3). 

The one-way ANOVA analysis demonstrated no significant between-groups differences when comparing the BISFed TFS scores (*p* = 0.892). However, significant differences were found for the MRE variable between sports classes in the composite static posturographic condition (*p* = 0.004; dg = 1.06, large) and with each of the individual static posturographic conditions (SNVF and SVF) (see Table 4). In addition, although nonsignificant differences were obtained across the single dynamic posturographic tasks, moderate effect sizes were also obtained in the composite dynamic performance (*p* = 0.17; dg = 0.55, moderate). Table 4 also shows the number of participants who were able to perform the whole test battery, revealing more difficulties in the dynamic test battery for the participants.

### 3.3. Sensitivity of the BISFed TFS

The ROC analyses were only applied to the composite static score, revealing that the optimum cut-off score was 2.61 mm (AUC: 0.726, 95% confidence interval (CI): 0.537–0.915; sensitivity: 69.2%; 1-specificity: 26.1%). Those scores suggest a worthless discriminative capacity of the posturographic static conditions to discriminate between BC1 and BC2 trunk function. 

## 4. Discussion

This study investigated if the current methods to assess trunk control in Boccia players with severe-to-moderate CP provide reliable, valid and sensitivity measures for classification purposes.

Regarding the inter-rater reliability of the BISFed TFS, the percentage of agreement between testers was very high, reaching an almost perfect level of agreement. However, it should be noted that the original scale is not a ratio scale, being one of the requirements requested by the International Paralympic Committee at the time of developing evidence-based classification [18]. For this study, authors converted the BISFed TFS evaluation judgements into a ratio-scale score, so there is an urgent need to include or look for other scales, such as the trunk control measurement scale [19], both validated for young people with cerebral palsy, to assess trunk function in Boccia players. 

Concerning the BISFed TFS concurrent validity, low-to-moderate correlations were obtained for two of the dynamic posturographic tasks (i.e., medial-lateral and circular) and for the composite dynamic score. These correlations seem to indicate that the BISFed TFS is more in line with a more dynamic trunk assessment rather than static trunk control. In fact, when players are asked to bend the trunk in the frontal and the transverse planes as much as possible, to return afterwards to the starting point (i.e., mid-line) without any external support [20], what is being measured is the limit of stability or dynamic control instead of static trunk control. 

Regarding the sensitivity of the BISFed TFS, our results suggest that this tool is not sensitive enough to discriminate between player BC1 and BC2 sport classes. These results can be construed from different perspectives. On the one hand, the allocation of a sport class in Boccia is not based solely on players´ trunk function, as there are other factors to consider, such as arm function or manual dexterity [21], that usually have greater importance for the classifiers for decision-making due to the high impact on performance (i.e., ball throwing). This rationale would be supported by the fact that dynamic posturographic tasks were also unable to discriminate between BC1 and BC2 classes. The low relevance of dynamic trunk control to be classified as BC1 and BC2 also seems supported by a potential floor effect observed in the dynamic tasks, which was more evident in the BC2 group than in the BC1. The Boccia players with the highest impairment present a severe trunk muscular weakness and altered selective motor control [22], which hinders them from keeping a vertical position out of the wheelchair and dynamically controlling the trunk [23]. From a clinical point of view, being able to accomplish the dynamic tasks could be reasonably used as a “cut-off” point when evaluating different levels of trunk functionality (i.e., more or less impaired) in this population. In this sense, whether dynamic trunk control was a relevant factor to classify Boccia players in BC1 and BC2 sport classes, most of the players unable to accomplish the dynamic posturographic task would mainly be grouped in the BC1 sport class. However, in our study, more BC2 players (27.8%; 10 participants) were unable to accomplish all dynamic tasks than BC1 players (15.4%: two participants), which reinforces the low relevance of dynamic trunk control to classify players in BC1 and BC2 sport classes.

On the other hand, although the BISFed TFS and dynamic posturographic tasks did not show significant differences between the sports classes included in this study, the posturographic test battery did so, but only in the static tasks. If we compare these results with the demands for this para-sport, we can notice that, in Boccia, as a precision sport, it is not necessary to perform many dynamic movements. Boccia requires that players throw balls accurately to get them as close as possible to the white ball (i.e., the jack), so they may not require broad trunk movements but small adjustments to stabilize themselves and achieve the most efficient throwing position [24]. While the BISFed TFS would be used to assess a trunk function dimension (i.e., the limit of stability), it would be possible that this capability is not needed in the Boccia-throwing performance and, therefore, not pertinent for classification purposes. The absence of between-group differences for the BISFed TFS could also be related to the lack of standardization of this protocol in the classification practice. While the posturographic test battery contemplates the standardization of the player´s sitting position, providing two fixation points (in the pelvis and in the ankles), the BISFed TFS provides none. The posturographic test battery appears to be more similar to Boccia in this aspect, as Boccia players are allowed to use some external support aid (i.e., straps or armrest) in order to improve their motor ability, as it happens in other para-sports [25]. Therefore, the fixation of certain points helps players to freeze degrees of freedom (i.e., compensatory strategies) benefiting the function of the trunk, which seems to be crucial to discriminate between different trunk function levels, especially in moderate-to-severe levels of impairment (i.e., it makes the protocol more sensitive to discriminate better between different performance profiles). 

Given the importance that the International Paralympic Committee (IPC) points in finding measuring instruments that are based on scientific evidence and sport-specific [18], the authors of this study urge work into the search for a field test that measures static trunk control according to the specific demands of this para-sport [25,26], measuring players’ trunk control functions in similar conditions to those used during Boccia competition. Currently, within all Paralympic sports, only for seated throwing [27] and wheelchair racing [28] in para-athletics and wheelchair rugby [29] are provided evidence-based and sport-specific methods to assess para-athletes´ trunk function. However, these studies evaluated trunk strength in non-CP para-athletes and required always dynamic trunk movements. Authors of this study suggest the use of accelerometers embedded into smartphones, which is an increasingly common method to quantify postural control in field settings due to its easy use, low cost and objectivity of measurements [30,31]. 

Some limitations of this study should also be noted. First, the participants of this study have competed in Boccia for several years with confirmed sport classes (i.e., BC1 or BC2), considering they are correctly classified. Second, although a larger sample in this study would have been desirable, players with such severity of impairment present more difficulties with transportation, constraining their recruitment for research. Third, further research would explore the potential differences between types of CP impairment, including a balanced proportion of para-athletes with spasticity, dyskinesia and/or mixed profiles. Finally, evaluating players with a higher level of performance would be also recommended (i.e., international level).

## 5. Conclusions

This study highlighted that the current practice to assess trunk control in Boccia players with severe-to-moderate CP might not be adequate in terms of validity and sensitivity to group players with more or less impaired trunk control. In the first instance, the BISFed TFS has shown a poor relationship with a posturographic laboratory test, besides not presenting a good ability to differentiate and, therefore, classify between players with different levels of trunk function. In addition, although the BISFed TFS has good inter-rater reliability, it seems to measure a trunk function more related to the limit of stability instead of trunk control. Finally, this study has shown that dynamic trunk control would not be as relevant as static trunk control is, demonstrating that trunk control evaluated through the BISFed TFS is not sport-specific for classification purposes in Boccia.

## Figures and Tables

**Table 1:** Participants’ demographic data.

Demographic Variables	BC1 *n* = 13	BC2 *n* = 23	Overall *n* = 36
Age (year)	39.00 ± 13.20	34.50 ± 14.14	36.33 ± 13.69
Body Mass (kg)	53.65 ± 10.96	51.56 ± 11.89	52.02 ± 11.04
Trunk Height (cm)	43.50 ± 2.12	54.67 ± 7.36	52.64 ± 8.02
Impairment type (muscle tone)			
Spasticity	7 (53.85)	13 (56.52)	20 (55.56)
Athetosis/Dystonia	1 (7.69)	4 (17.39)	5 (13.89)
Mixed (spastic/athetoid)	5 (38.46)	6 (26.09)	11 (30.56)
Sex			
Male, *n* (%)	6 (46.15)	14 (60.87)	20 (55.56)
Female, *n* (%)	7 (53.85)	9 (39.13)	16 (44.44)
Competition Level			
Regional, *n* (%)	9 (69.23)	10 (43.48)	19 (52.78)
National, *n* (%)	3 (23.08)	11 (47.83)	14 (38.89)
International, *n* (%)	1 (7.69)	2 (8.69)	3 (8.33)
GMFCS (level), median (range)	4 (4–4)	4 (2–4)	4 (2–4)
Level II, *n* (%)	--	6 (26.09)	6 (16.67)
Level III, *n* (%)	--	5 (21.74)	5 (13.89)
Level IV, *n* (%)	13 (100)	12 (52.17)	25 (69.44)

GMFCS = gross motor functional classification scale, M ± SD.

**Table 2:** Classifiers’ profiles that conducted Boccia International Sports Federation (BISFed) trunk function scale (TFS) assessments.

Demographic Variables	Classifier/Tester #1	Classifier/Tester #2
Age (year)	48	39
Sex	Female	Male
Background	Physiotherapist	Sports Sciences PhD
BISFed Classification Level	Level 3	Level 3
Classification Experience in Boccia		
International (year)	12	6
National (year)	22	16
Experience in other Paralympic sports	Cerebral Palsy Football	Cerebral Palsy Football
	World Para Athletics	World Para Athletics
	Para Equestrian	Wheelchair Slalom

**Table 3:** Correlations between the BISFed TFS and the posturographic test battery.

	BISFed TFS	SNVF	SVF	DML	DAP	DC	Comp. Static	Comp. Dynamic
BISFed TFS	--	−0.23	−0.31	−0.38 *	−0.35	−0.46 *	−0.27	−0.51 **
SNVF			0.85 **	0.46 **	0.53 **	0.40 *	0.97 **	0.61 **
SVF				0.39 *	0.54 **	0.36	0.95 **	0.58 **
DML					0.90 **	0.73 **	0.45 **	0.94 **
DAP						0.79 **	0.56 **	0.96 **
DC							0.40 *	0.92 **
Comp. Static								0.63 **
Comp. Dynamic								

* Significant Pearson correlation at *p* < 0.05, ** Significant Pearson correlation at *p* < 0.01, BISFed TFS = Boccia International Sports Federation trunk functional scale, SNVF = static without visual feedback, SVF = static with visual feedback, DML = dynamic medial-lateral with visual feedback, DAP = dynamic anterior-posterior with visual feedback, DC = dynamic circular with visual feedback and Comp. = composite.

**Table 4:** Descriptive statistics and ANOVA (one factor) about mean radial error (MRE).

Variable	N_BC1_ + N_BC2_ = N	BC1 (Mean ± SD)	BC2 (Mean ± SD)	F	*p*	*d*_g_
BISFed TFS (unitless)	13 + 23 = 36	2.2 ± 1.1	2.3 ± 1.1	0.02	0.892	0.05
SNVF (mm)	13 + 23 = 36	7.9 ± 6.8	3.4 ± 2.0	8.93	0.005	1.01
SVF (mm)	13 + 23 = 36	6.2 ± 4.9	2.7 ± 2.3	8.43	0.006	0.99
DML (mm)	12 + 22 = 34	8.3 ± 4.7	7.9 ± 4.3	0.06	0.811	0.09
DAP (mm)	12 + 20 = 32	8.8 ± 4.3	7.5 ± 3.8	0.81	0.374	0.32
DC (mm)	11 + 15 = 26	11.7 ± 4.4	8.6 ± 4.5	2.96	0.098	0.67
Composite Static (mm)	13 + 23 = 36	7.0 ± 5.7	3.0 ± 1.8	9.62	0.004	1.06
Composite Dynamic (mm)	11 + 15 = 26	9.4 ± 4.1	7.3 ± 3.4	1.97	0.173	0.55

BISFed TFS = Boccia International Sports Federation trunk functional scale, SNVF = static without visual feedback, SVF = static with visual feedback, DML = dynamic medial-lateral with visual feedback, DAP = dynamic anterior-posterior with visual feedback, DC = dynamic circular with visual feedback and *d*g = effect size.
